# Low‐energy shock wave pretreatment recruit circulating endothelial progenitor cells to attenuate renal ischaemia reperfusion injury

**DOI:** 10.1111/jcmm.15678

**Published:** 2020-08-06

**Authors:** Jingyu Liu, Quanliang Dou, Changcheng Zhou, Liuhua Zhou, Feng Zhao, Luwei Xu, Zheng Xu, Yuzheng Ge, Ran Wu, Ruipeng Jia

**Affiliations:** ^1^ Department of Urology Nanjing First Hospital Nanjing Medical University Nanjing China

**Keywords:** C‐X‐C chemokine receptor type 7, stromal cell‐derived factor 1, endothelial progenitor cells, ischaemia reperfusion injury, low‐energy shock wave

## Abstract

Low‐energy shock wave (LESW) has been recognized as a promising non‐invasive intervention to prevent the organs or tissues against ischaemia reperfusion injury (IRI), whereas its effect on kidney injury is rarely explored. To investigate the protective role of pretreatment with LESW on renal IRI in rats, animals were randomly divided into Sham, LESW, IRI and LESW + IRI groups. At 4, 12, 24 hours and 3 and 7 days after reperfusion, serum samples and renal tissues were harvested for performing the analysis of renal function, histopathology, immunohistochemistry, flow cytometry and Western blot, as well as enzyme‐linked immunosorbent assay. Moreover, circulating endothelial progenitor cells (EPCs) were isolated, labelled with fluorescent dye and injected by tail vein. The fluorescent signals of EPCs were detected using fluorescence microscope and in vivo imaging system to track the distribution of injected circulating EPCs. Results showed that pretreatment with LESW could significantly reduce kidney injury biomarkers, tubular damage, and cell apoptosis, and promote cell proliferation and vascularization in IRI kidneys. The renoprotective role of LESW pretreatment would be attributed to the remarkably increased EPCs in the treated kidneys, part of which were recruited from circulation through SDF‐1/CXCR7 pathway. In conclusion, pretreatment with LESW could increase the recruitment of circulating EPCs to attenuate and repair renal IRI.

## 1 INTRODUCTION

Renal ischaemia reperfusion injury (IRI) is caused by an initial interruption of renal blood supply and subsequent blood reperfusion, which commonly occurs during the progress of multiple clinical settings (kidney transplantation, nephron‐sparing surgery, cardiovascular surgery, etc) and being the leading cause of acute kidney injury (AKI).^1^, ^2^ Renal IRI is characterized by the injury of renal tubular epithelial cells and microvascular endothelium, which can lead to the decrease of renal function,^3^, ^4^ and being a global public health concern associated with increasing morbidity, mortality and medical resource costs.^5^ Therefore, identifying potential therapeutic interventions for the treatment or restoration of renal IRI remains imperative.

High‐energy shock wave has been traditionally used for urinary stones more than 30 years, but shock wave‐induced kidney damage is unavoidable.^6^ In recent years, low‐energy shock wave (LESW) with an energy density of approximately 10% for urolithiasis has been confirmed to be a safe and effective treatment for certain diseases, such as musculoskeletal disorders,^7^ myocardial infarction^8^ and erectile dysfunction.^9^ Previous studies showed that LESW has special properties of promoting angiogenesis, anti‐inflammatory, suppressing oxidative stress in fascia, myocardium, bladder and limb.^7^, ^8^, ^10^, ^11^ However, the role of LESW in renal IRI is rarely reported and the possible mechanism needs further exploration.

Stem or progenitor cell therapy was proved as an effective approach to improve renal function after renal ischaemia.^12^ Several studies documented that endothelial progenitor cells (EPCs) can attenuate renal injury, possibly by increasing neovascularization and inhibiting inflammation and oxidative stress.^13^, ^14^, ^15^ Evidence revealed that pretreatment towards the kidney could remarkably improve the number of EPCs in renal tissues.^15^, ^16^, ^17^ However, the source of increased EPCs in kidney after preconditioning, being either inherent progenitor cells proliferation or circulating EPCs homing, has not been further clarified. EPCs are equipped with receptors (C‐X‐C chemokine receptor type 4 (CXCR4) and C‐X‐C chemokine receptor type 7 (CXCR7)) for SDF‐1, which can allow them to homing from circulation to target tissue.^18^ Our previous research found that ischaemia preconditioning could increase the expression of SDF‐1 and attenuate partial nephrectomy‐induced IRI.^17^ A research also reported that SDF‐1 regulated the migration, proliferation and tube formation of rat bone marrow–deprived EPCs through CXCR7.^19^ CXCR7 also play an important role in improving the function of EPCs and EPCs with low levels of CXCR7 could lead to impaired angiogenesis and re‐endothelialization in hypertensive patients.^20^, ^21^ SDF‐1/CXCR7 pathway may play an important role in the recruitment of EPCs.

Therefore, we observed the effect of preconditioning with LESW on kidney IRI in a rat model, and further explored the potential role and mechanism of LESW in circulating EPCs homing.

## 2 MATERIALS AND METHODS

### 2.1 Animals

All animals were bred and housed in the Experimental Animal Center of Nanjing First Hospital. All procedures were approved by the Ethics Committee for the Use of Experimental Animals of the Nanjing First Hospital, Nanjing Medical University. The investigation was conducted according to the Guide for the Care and Use of Laboratory Animals of the National Institutes of Health (NIH). Male Sprague Dawley rats weighing 250‐300 g were housed in a standard room under controlled conditions (suitable humidity and temperature and a 12‐hour dark/12‐hour light cycle) and with free access to food and water.

### 2.2 Experimental design and surgical procedures

The rats were anesthetized with sodium pentobarbital (50 mg/kg, ip). After back skin preparation, a length 1‐2 cm right back incision was made, and the right kidney was removed. During the surgery, a heat lamp was used to maintain at approximately 37°C and body temperature was monitored by rectal probe.

After two weeks, the rats were randomly divided into four groups. Group 1 was the sham group, in which the left renal artery was separated without clamping. Group 2 was the LESW group: after anaesthesia with sodium pentobarbital (50 mg/kg, ip), the hair on the left flank was removed. The rats in a prone position, the LESW probe, which generated shock wave through the acceleration of a projectile inside the device (DolorClast, EMS, Swiss) and used the following parameters: 0.1 mJ/mm^2^ energy flux density at 1 Hz for 200 impulses,^22^ attached tightly to the left flank and a coupling agent was applied. Then, the left renal artery was separated without clamping. Group 3 was the IRI group, in which the left renal arteries were occluded using a non‐traumatic vascular clamp for 45 minutes as previously described.^23^ Group 4 was the LESW + IRI group. In this group, after the procedure of LESW as described in Group 2, the left renal arteries were also occluded using a non‐traumatic vascular clamp for 45 minutes. The incisions were sutured in all groups after surgery. To explore the effect of LESW preconditioning on the recruitment of circulating EPCs after renal IRI, another rats injected with labelled circulating EPCs (2 × 10^6^) via tail vein were randomly assigned into four groups as described above. To evaluate the potential effect of SDF‐1/CXCR7 pathway on the homing of circulating EPCs after renal IRI, rats were randomly assigned as follow: (a) IRI group, the processing method as described in group 3; (b) LESW + IRI group, the procedure as described in group 4; (c) LESW + IRI + CCX771 group, CCX771 administration (ChemoCentryx; 25 mg/kg, intraperitoneal injection, daily for a week) before pretreatment with LESW.

### 2.3 Blood and kidney tissue preparation

At 4, 12, 24 hours and 3 and 7 days after reperfusion, a midline incision was made under complete anaesthesia and 5 mL of blood was collected from the abdominal inferior vena cava and centrifuged at 1600 *g* for 15 minutes. The supernatants were collected and store at −80°C for further testing. The rats were killed and perfused with 0.9% saline. The left kidneys were harvested for subsequent experiments.

### 2.4 Renal function analysis

The supernatants were tested to measure serum creatinine (SCr), cystatin C (Cys C) and blood urine nitrogen (BUN) levels by clinically automated analysis methods (Hitachi 7600‐10).

### 2.5 Histological and immunohistochemical analysis

To assess the morphological integrity, the harvested tissues at 24 hours after reperfusion were fixed in paraffin, sectioned and analysed by haematoxylin and eosin staining (H&E). Histological score of the kidney (HSK) was evaluated in a blind manner by two experienced pathologists. As described previously,^2^ three fields per section were scored on a scale of 0‐4 (0, 0%; 1, 0%‐5%; 2, 5%‐25%; 3, 25%‐75%; and 4, 75%‐100%). The effect of LESW on apoptosis, cell proliferation and small vascular density was observed at 3 days after reperfusion. Apoptosis was evaluated with terminal transferase–mediated deoxyuridine triphosphate nick‐end‐labelling (TUNEL) assay (Roche) according to the manufacturer's protocol. Proliferation of renal cell was detected by using antiproliferating cell nuclear antigen (anti‐PCNA) antibody (Abcam). Small vascular density was measured by anti‐CD34 antibody (Abcam). Anti‐SDF‐1 antibody (Abcam) was used to observe the cell types that express SDF‐1 at 24 hours after reperfusion. Immunohistochemical assays for the staining of anti‐PCNA, anti‐CD34 and anti‐SDF‐1 were performed according to our previous protocol.^23^

### 2.6 Flow cytometric analysis

To quantify EPCs in IRI kidneys, flow cytometry was introduced to identify CD34, CD133 and VEGFR2 triple‐positive cells.^24^ In brief, kidney tissues were homogenized using a glass homogenizer on the white ice, and then, the suspension was filtered through 75‐μm mesh and separated using density gradient centrifugation. The middle mononuclear cells were collected and further stained with anti‐CD133‐PE (Bioss), anti‐CD34‐FITC (Bioss) and anti‐VEGFR2‐APC (Bioss) for 30 minutes at room temperature. Then, cells were analysed with FACSCalibur (BD Bioscience). An isotype‐matched IgG was used as a negative control for each primary antibody.

### 2.7 Western blot analysis

Total proteins were extracted with a protein kit (Solarbio). The protein samples were denaturalized in boiling water. Equal amount of protein samples was loaded on each lane and separated on SDS‐polyacrylamide gel electrophoresis and transferred to polyvinylidene difluoride membranes (EMD Millipore). Afterwards, the membranes were blocked with 5% non‐fat milk and incubated with primary antibodies against SDF‐1 (Abcam) and CXCR7 (Abcam) over night at 4°C. Then, the membranes were rinsed with Tris‐buffered saline Tween‐20 (TBST) thrice, incubated with a secondary horseradish peroxidase‐conjugated antibody (Cell Signaling) for 2 hours at room temperature. Immunoblot signals were detected by an enhanced chemiluminescence system (ECL kit) and quantified by scanning densitometry using the ImageJ analysis system (NIH).

### 2.8 Isolation and identification of EPCs

To assess the effect of LESW preconditioning on circulating EPCs homing, we extracted circulating EPCs from rat peripheral blood and tracked its distribution in ischaemic kidneys. Briefly, blood samples were diluted 1:1 with phosphate‐buffered saline (PBS, Gibco), overlaid onto an equal volume of Ficoll‐Paque (1.084 g/mL, GE Healthcare) and then centrifuged at 1100 *g* for 30 minutes at room temperature. The buffy coat from the middle layer was transferred, and the isolated mononuclear cells (MNC) were suspended to a concentration of 1 × 10^6^ cells/mL in complete endothelial cell growth medium (EGM)‐2 (Gibco) with 10% foetal bovine serum (FBS, Gibco). The suspension was seed into 6‐well plates precoated with type Ⅰ rat tail collagen (Corning) and incubated at 37°C in a humidified 5% CO_2_ incubator. After 5‐7 days of incubation, non‐adherent cells and debris were discarded and the fresh EGM‐2 was added. The medium was replaced every 3‐4 days. Primary cells reached 80%‐90% confluence were purified in accordance with the different sensitivity of various adherent cells to trypsin, and subcultured in the ratio of 1:2 for subsequent experiments.

Subcultured cells were washed three times with PBS and incubated with 10 µg/mL Dil‐acetylated low‐density lipoprotein (Dil‐Ac‐LDL, Solarbio) for 4 hours. Then, the cells were washed and fixed in 4% prechilled paraformaldehyde for 15 minutes. After washes, the fixed cells were incubated for 1 hour with 10 µg/mL fluorescein isothiocyanate–labelled Ulex europaeus agglutinin‐I (FITC‐UEA‐I, Sigma‐Aldrich). Nuclei were stained with 4,6‐diamidino‐2‐phenylindole (DAPI, Beyotime). Three fields were randomly counted to evaluate the numbers of double‐positive staining EPCs using a fluorescence microscope (Olympus). Moreover, subcultured and purified cells were also identified by flow cytometric analysis with a panel of cell‐specific antibodies: anti‐CD133‐PE (Bioss), anti‐CD34‐FITC (Bioss), anti‐CD31‐FITC (Bioss), anti‐VEGFR2‐FITC (Bioss), anti‐CD45‐FITC (Bioss) and anti‐CD14‐PE (Bioss). An isotype‐matched IgG was used as a negative control for each primary antibody. We also identified the subcultured cells with immunofluorescence. Briefly, the cultured cells were incubated with primary antibodies anti‐CD31 (Abcam), anti‐133 (Abcam), anti‐CD31 (Abcam) and anti‐VGFR2 (Abcam) over night at 4°C and washed with PBS thrice, then incubated with a secondary antibody Alexa Fluor 488 or 555 (Bioss) at room temperature. Nuclei were stained with DAPI.

### 2.9 Cell labelling and tracking

EPCs were labelled with both the CellTracker™ CM‐Dil and Dil‐C_18_(5)‐DS (Molecular Probes) before tail vein injection according to the manufacturer's instruction. Briefly, EPCs were incubated with CM‐Dil (2 μg/mL) and Dil‐C_18_(5)‐DS (2 μg/mL) for 5 minutes at 37°C and following 15 minutes at 4°C, and washed twice before tail vein injection. The labelling efficiency of CM‐Dil and Dil‐C_18_(5)‐DS was verified using fluorescence microscope and FACSCalibur, respectively. Labelled EPCs (2 × 10^6^) were injected by using a previously described protocol with minor modifications,^25^ and their fate and distribution in kidneys were investigated by IVIS Spectrum small‐animal in vivo imaging system (PerkinElmer) and fluorescence microscope. Briefly, the kidneys of rats injected with EPCs were collected 24 hours after reperfusion. Following a detection by the in vivo imaging system, the kidneys were cryopreserved in OCT compound (Tissue‐Tek), followed by preparation of 5‐μm cryosections, fixation in acetone for 2 minutes and blocking for 30 minutes in PBS + 3% bovine serum albumin (BSA, Biofroxx). Then, sections were incubated with anti‐CD133 (Abcam) and DAPI. Subsequently, fluorescence microscope (Olympus) was adopted to monitor CM‐Dil (red) and CD133 (green) double‐positive cells.

### 2.10 Enzyme‐linked immunosorbent assay

Levels of SDF‐1 in serum and kidney tissues at different time points after reperfusion in the four groups were determined by enzyme‐linked immunosorbent assay (ELISA) kit (Elabscience) according to the manufacturer's instructions. The absorbance was evaluated on a microplate reader (Tecan), and the concentration was calculated on the basis of a standard curve. The level of SDF‐1 in kidney tissue was determined as previously described.^23^ Protein content was collected from the supernatant of kidney homogenate. Results were normalized to the total protein concentration of supernatant for further comparison. This experiment was performed in triplicate.

### 2.11 Migration assay

A 24‐well Boyden chamber with an 8‐μm pore size polycarbonate membrane (Corning) was used to detect EPCs migration. Briefly, 5 × 10^4^ cells were seeded in the top chamber of the transwell, whereas the lower chamber was filled with culture medium supplemented with SDF‐1 (0, 10, 25, 50, 100, 200 ng/mL and 200 ng/mL + CCX771 1 μg/mL, Sigma‐Aldrich), and CCX771 was added to both chambers 2 hours before adding SDF‐1 to the lower chamber. Cells were cultured for 24 hours at 37°C in a well‐humidified incubator, and then the cells located on lower surface were fixed by formaldehyde for 15 minutes, stained with 0.1% crystal violet. The cells that migrated to the lower surface were quantified by counting under a microscope with magnification of 200× in five different predetermined fields.

### 2.12 Statistical analysis

Results were expressed as mean ± standard deviation (SD). Statistical differences between groups were evaluated by one‐way ANOVA. The Tukey test was applied for post hoc comparisons. *P* < .05 was considered statistically significant. All statistical analysis was performed with SPSS software (version 21.0; SPSS Institute).

## 3 RESULTS

### 3.1 Effects of LESW pretreatment on renal function

**Figure 1:**
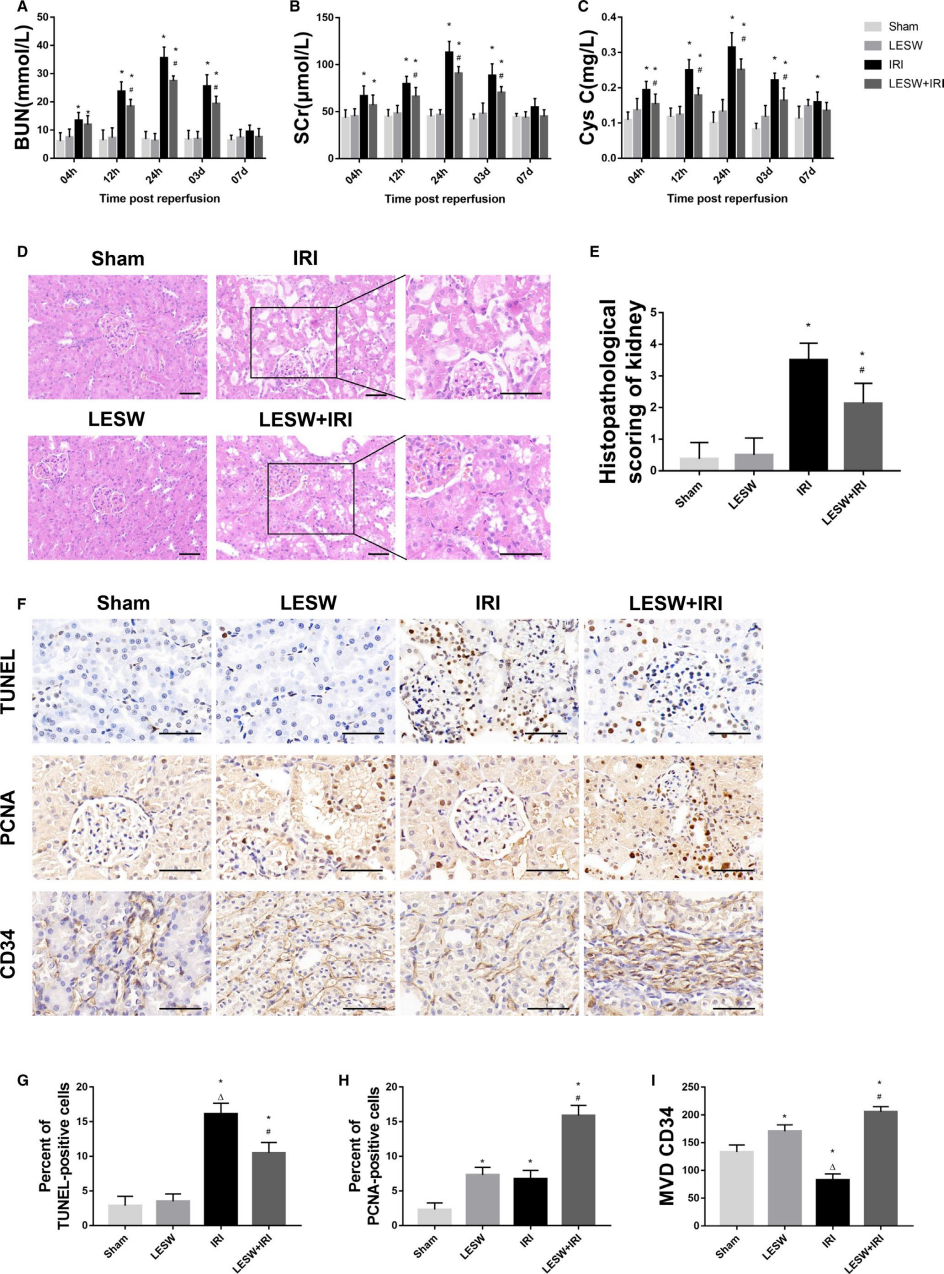
Pretreatment with LESW attenuated renal injury and promoted kidney repair. A‐C, BUN, SCr, and Cys C were evaluated at 4, 12, 24 h, and 3, 7 d after reperfusion in the four groups. D, Representative images of H&E staining preformed on sections of kidneys at 24 h after reperfusion in the four groups. E, Histopathological scoring of kidney at 24 h after reperfusion in the four groups. F, Representative images of TUNEL, PCNA, and CD34 staining in kidneys at 3 d after reperfusion in the four groups. G‐I, Semi‐quantitative analysis of TUNEL‐positive cells, PCNA‐positive cells, and MVD CD34 were performed by using ImageJ software. Scale bar = 50 μm. *P < .05 (LESW, IRI, LESW + IRI vs Sham); ^#^ *P* < .05 (LESW + IRI vs IRI); ^Δ^ *P* < .05 (IRI vs LESW). BUN, blood urea nitrogen; Cys C, cystatin C; H&E, haematoxylin and eosin; IR, ischaemia reperfusion; LESW, low‐energy shock wave; MVD, microvessel density; PCNA, proliferating cell nuclear antigen; SCr, serum creatinine; TUNEL, terminal transferase‐mediated deoxyuridine triphosphate nick‐end‐labelling

Compared with Sham group and LESW group, the renal function of animals suffered from IRI showed a significant deterioration, as indicated by the changes of BUN, SCr, Cys C (Figure 1A‐C). These renal function markers changed in a single phase: a significant increase at 4 hours, a peak at 24 hours and a baseline value at 7 days. These changes were significantly attenuated in the LESW preconditioned rats than that in the IRI group. Moreover, compared with the Sham group, LESW intervention merely slightly altered renal function in non‐renal ischaemia rats without significant difference.

### 3.2 Effects of LESW pretreatment on renal tubular injury

Histopathological evaluation of renal tissue revealed severe acute tubular injury in the kidneys at 24 hours after reperfusion in the IRI group and the LESW + IRI group. These features included tubular epithelial cell necrosis, tubular dilatation and/or atrophy, inflammatory cell infiltration and cellular and interstitial oedema, which were less pronounced in the LESW + IRI group. Higher HSK could be observed in the kidneys of IRI group than that in the LESW + IRI group at 24 hours after reperfusion. There were no significant differences in the HSK between the Sham group and the LESW group (Figure 1D,E).

### 3.3 Effects of LESW pretreatment on cell apoptosis and proliferation, and microvasculature in kidney tissues after IRI

The effect of LESW administration on tubular cell loss was observed by assessing cell apoptosis in the kidney at 3 days after reperfusion. Rats suffered renal IRI showed a significant increase in the proportion of TUNEL‐positive cells compared with the Sham group and the LESW group. Treatment with LESW before ischaemia could significantly reduce the proportion of TUNEL‐positive cells. Rats received only the pretreatment with LESW exhibited a slight increase in the proportion of TUNEL‐positive cells compared with the Sham group (Figure 1F,G). Consistent results were also observed by evaluating PCNA expression in the kidney. The proportion of PCNA‐positive cells in renal sections were elevated in animals that suffered IRI, whereas the increase was significantly higher in the LESW + IRI group compared with the IRI group (Figure 1F,H). Additionally, the detection of endothelial cell marker CD34, an indicator of the microvessel density (MVD), illustrated a similar trend among the four groups (Figure 1F,I).

### 3.4 LESW pretreatment increased EPCs in IRI kidneys

**Figure 2:**
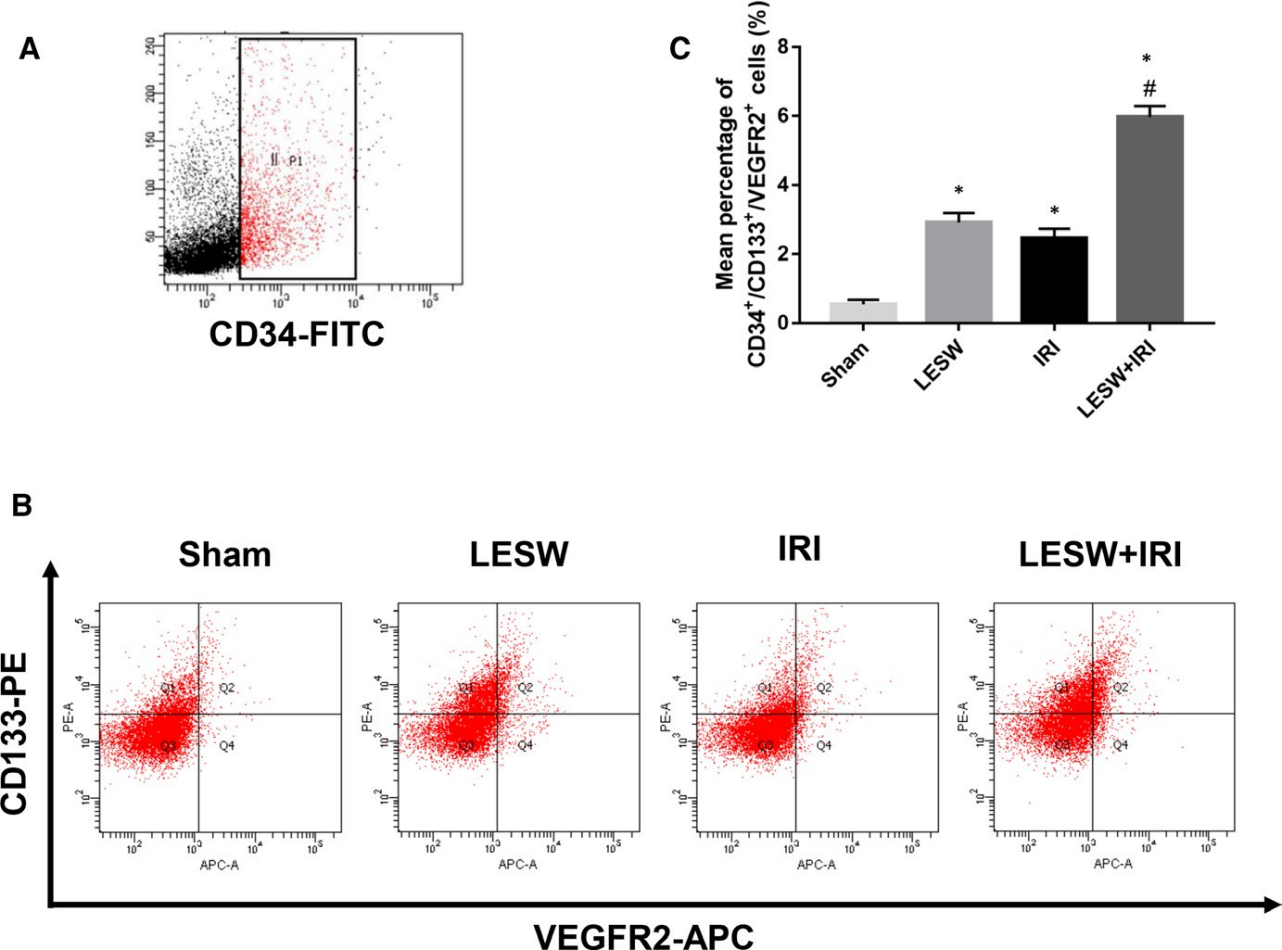
LESW increased the number of endothelial progenitor cells (EPCs) in kidney tissues. A and B, The typical EPCs phenotype of isolated EPCs in the kidney was confirmed by CD34, CD133, and VEGFR2 by flow cytometry in the four groups. Representative flow cytometry histograms of isolated EPCs. C, Quantitative analysis of CD34^+^/CD133^+^/VEGFR2^+^ cells in the four groups. *P < .05 (LESW, IRI, LESW + IRI vs Sham); ^#^ *P* < .05 (LESW + IRI vs IRI); LESW, low‐energy shock wave; VEGFR2, vascular endothelial growth factor receptor 2

To determine the number of EPCs in kidney tissues, we freshly isolated renal EPCs from four groups. Results showed CD133^+^/CD31^+^/VEGFR2^+^ cells were increased in the groups suffered IRI or independent LESW compared with the Sham group, and a significant increase of the triple‐positive cells could be identified in the LESW + IRI group than that in the IRI group (Figure 2).

### 3.5 Characterization of circulating EPCs from peripheral blood

**Figure 3:**
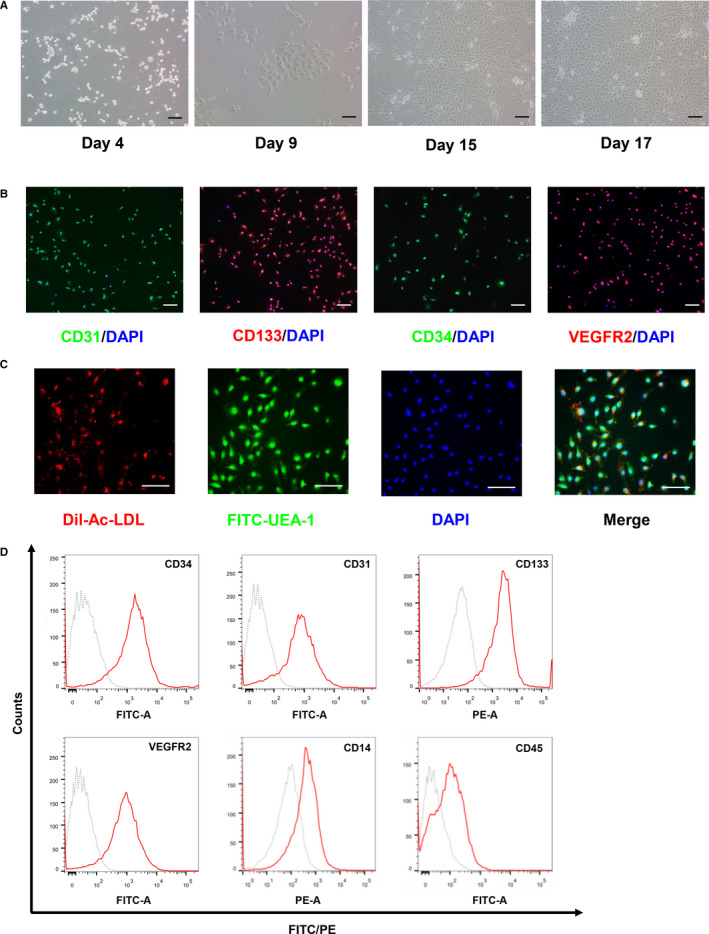
Characterization of isolated circulating endothelial progenitor cells (EPCs). A, Cells adhered to culture plates at day 4. Spindle‐like cells appeared at day 9. Typical cobblestone‐like cells appeared at day 15. Cells emerged after day 17 of cultivation. B, Representative images of immunofluorescent staining using EPCs specific markers CD31/CD133/CD34/VEGFR2 and nuclear marker DAPI. C, Dil‐Ac‐LDL and FITC‐UEA‐1 uptake assay showed the both Dil‐Ac‐LDL/FITC‐UEA‐1‐positive cells, which indicated that the cultured cells were EPCs. D, Representative flow cytometry histograms of cultured EPCs. Scale bar = 50 μm. DAPI, 4′,6‐diamidino‐2‐phenylindole; Dil‐Ac‐LDL, 1,1′‐dioctadecyl‐3,3,3′,3′‐tetramethylindocarbocyanine acetylated low‐density lipoprotein; FITC‐UEA‐1, fluorescein isothiocyanate Ulex europaeus agglutinin‐1; VEGFR2, vascular endothelial growth factor receptor 2

To confirm whether the pretreatment with LESW could recruit EPCs from circulating, MNC were isolated from peripheral blood by Ficoll density gradient centrifugation. After primary cultures were incubated for 4 days, small numbers of cells adhered to collagen Ⅰ‐precoated culture plates and spread out. After 9‐15 days in complete EGM‐2 medium, numerous cell clusters appeared that showed clonal growth and exhibited typical cobblestone‐like morphology under inverted phase‐contrast microscopy. Cells reach 80%‐90% confluence after approximately 17 days of primary culture (Figure 3A). After purifying and subculture, EPCs at passage 3 were stained with the EPCs specific markers CD31, CD133, CD34 and VEGFR2, and almost all cells were positive for these four markers under fluorescence microscope (Figure 3B). The following quantitative analysis using flow cytometry also validated the above consequences and further revealed the negative for CD14 and CD45 (Figure 3D). Additionally, we also found that the cultured cells could incorporate Dil‐Ac‐LDL and bind Fitc‐UEA‐1 (Figure 3C).

### 3.6 LESW pretreatment enhanced the recruitment of circulating EPCs

**Figure 4:**
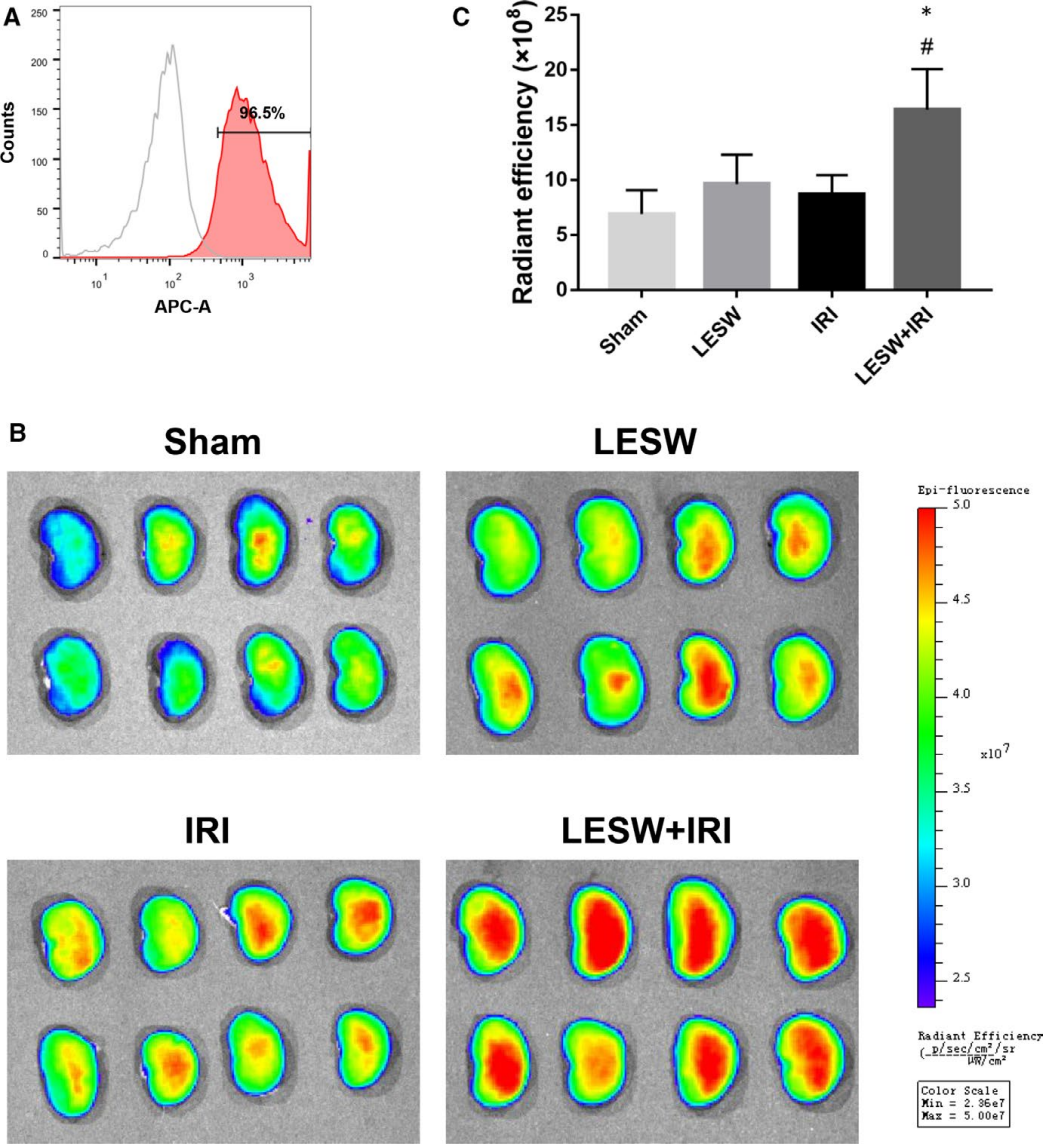
The distribution of Dil‐C_18_(5)‐DS labelled circulating endothelial progenitor cells (EPCs). A, Representative flow cytometry histograms of Dil‐C18(5)‐DS label ratio. B, Representative fluorescence images of kidneys detected by in vivo imaging system. C, Quantitative analysis of the retention of the Dil‐C_18_(5)‐DS labelled circulating EPCs in kidneys at 24 h after reperfusion in the four groups. *P < .05 (LESW + IRI vs Sham); ^#^ *P* < .05 (LESW + IRI vs IRI). LESW, low‐energy shock wave

**Figure 5:**
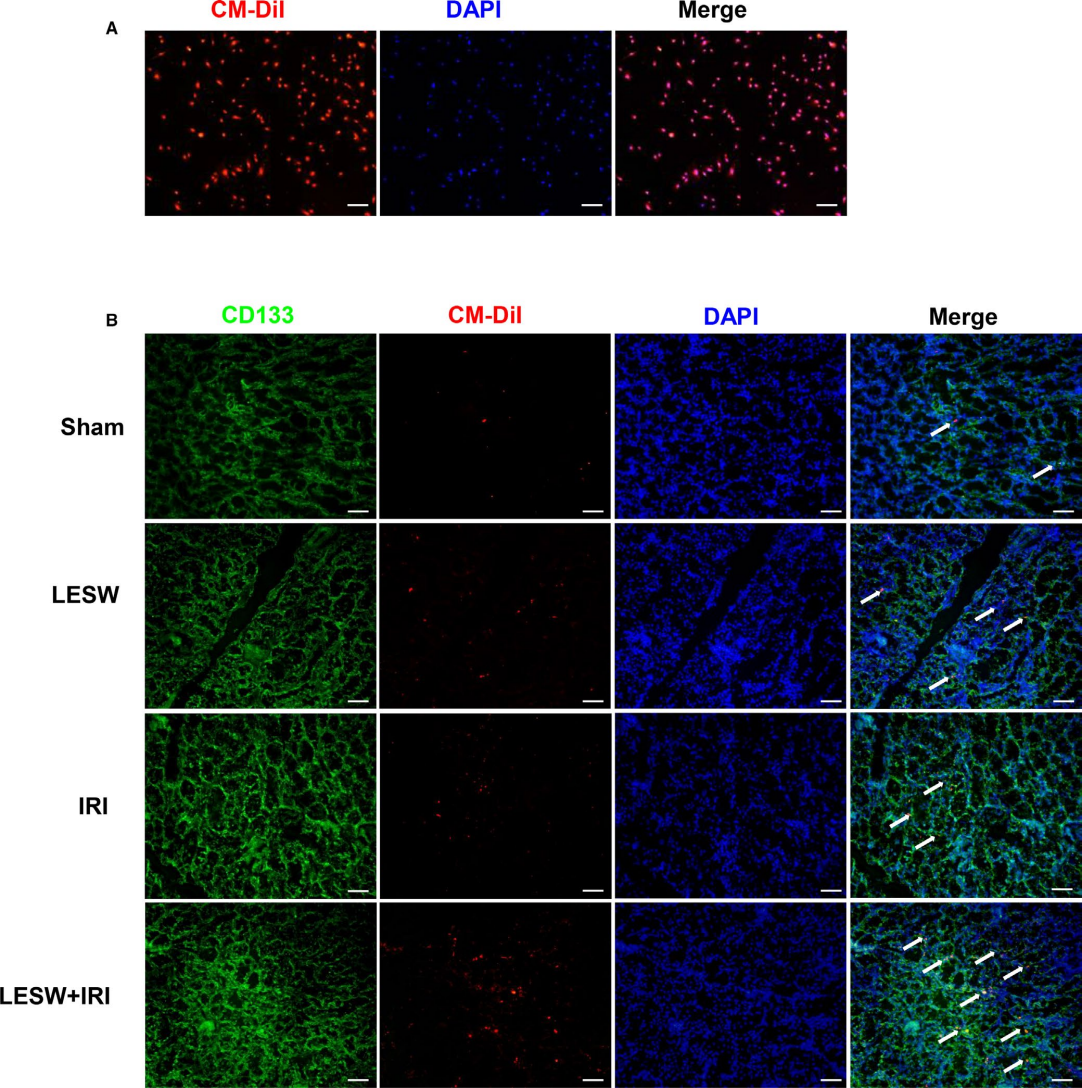
Cell tracking in rats injected with CM‐Dil labelled circulating endothelial progenitor cells (EPCs). A, Representative images of CM‐Dil labelled isolated circulating EPCs. B, Representative images of CD133^+^/CM‐Dil^+^ cells in kidneys at 24 h after reperfusion in the four groups. Scale bar = 50 μm. LESW, low‐energy shock wave

To explore the source of increased EPCs in kidneys after IRI and elucidate the potential promotion effect of LESW on circulating EPCs homing, the rats in each group were intravenously injected with labelled circulating EPCs. The flow cytometry and fluorescence examination of the disposed cells confirmed that almost all circulating EPCs were successfully marked with Dil‐C18(5)‐DS and CM‐Dil (Figures 4A, 5A). At 24 hours after reperfusion, the harvested kidneys and following sections were analysed using the in vivo imaging system and fluorescence microscope, respectively. The analysis of entire kidney by in vivo imaging revealed that the labelled circulating EPCs were present in all kidneys, whereas the fluorescence intensity in the LESW + IRI group was significantly higher than that in the other three groups, suggesting the highest number of labelled circulating EPCs in the LESW + IRI group (Figure 4B,C). Consistently, immunofluorescence staining of frozen sections demonstrated that the isolated and marked circulating EPCs could be recruited to the kidney tissues, and LESW preconditioning could remarkably increase CD133^+^/CM‐Dil^+^ cells in the IRI kidneys (Figure 5B).

### 3.7 Expression of SDF‐1 and CXCR7 in rats

**Figure 6:**
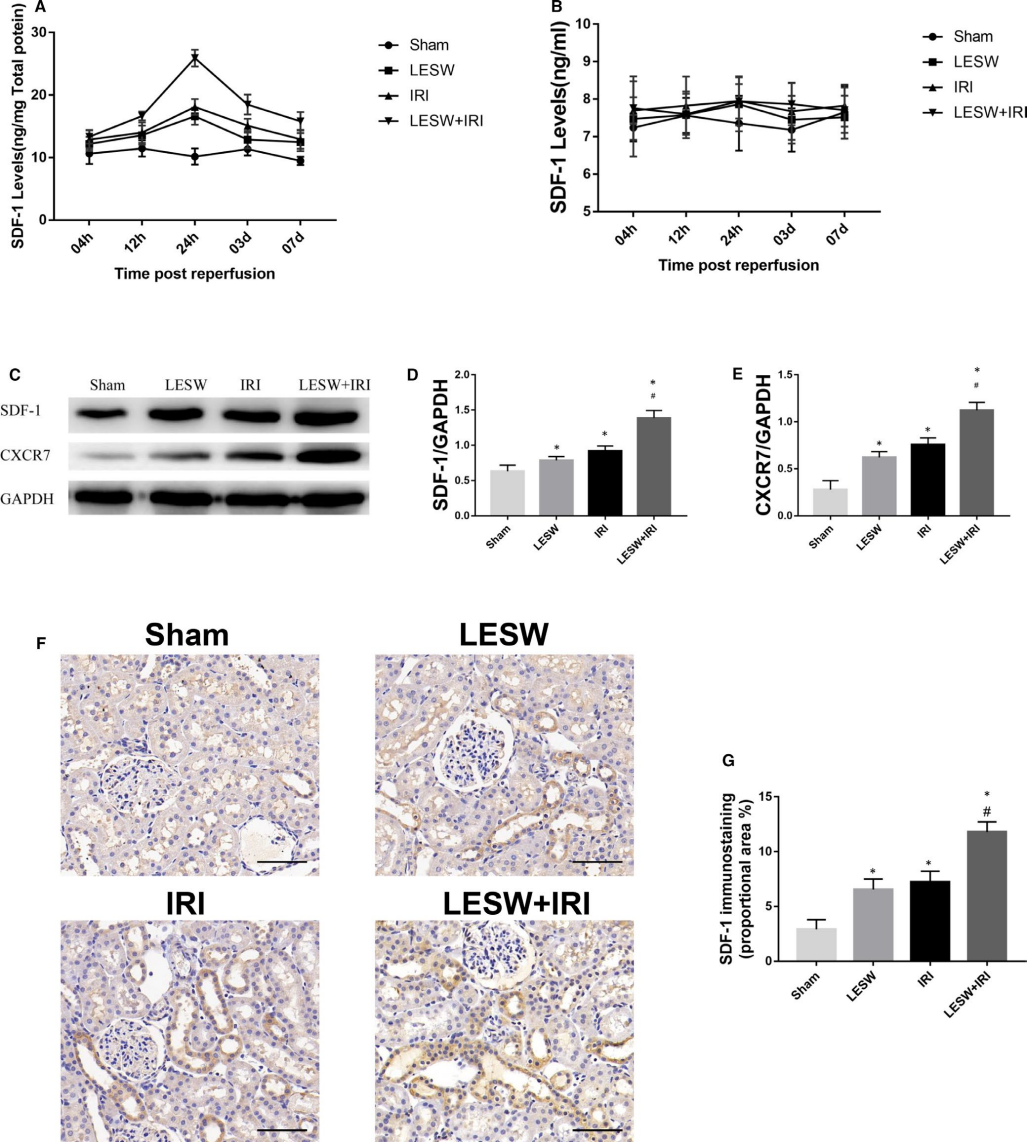
The expression of SDF‐1 and CXCR7 in four groups. A and B, SDF‐1 levels of kidneys and serum at different times after reperfusion in the four groups. C‐E, Relative abundance of SDF‐1/GAPDH and CXCR7/GAPDH were quantified according to Western blots at 24 h after reperfusion in the four groups. F, Representative images of SDF‐1 staining in kidneys at 24 h after reperfusion in the four groups. G, Semi‐quantitative analysis of SDF‐1 immunostaining proportional area. Scale bar = 50 μm. *P < .05 (LESW, IRI, LESW + IRI vs Sham); ^#^ *P* < .05 (LESW + IRI vs IRI). LESW, low‐energy shock wave

To detect the potential role of SDF‐1/CXCR7 pathway in LESW‐mediated EPCs homing, we investigated the expression of SDF‐1 in serum and the kidneys, and CXCR7 in the kidneys. The SDF‐1 levels of the kidneys in the LESW, IRI and LESW + IRI group were altered at different time points, and the difference reached a significant level at 24 hours after reperfusion when compared LESW + IRI group with the other three groups (Figure 6A). However, the levels of SDF‐1 in serum of each group revealed no significant fluctuations at different time points, and there was no significant difference between four groups (Figure 6B). Western blot analysis showed that LESW treatment and IRI could induce the up‐regulation of SDF‐1 in the kidneys, and the level of SDF‐1 was significantly heightened in the LESW + IRI group than that in the other three groups (Figure 6C,D). A similar trend could also be yielded when analysed the level of CXCR7 in the kidneys between these groups (Figure 6C,E). Meanwhile, the results of immunohistochemistry revealed that SDF‐1 was mainly expressed at the epithelial cells of the distal renal tubules and collecting ducts, and the expression of SDF‐1 in the LESW + IRI group was higher than that in the LESW and IRI group (Figure 6F,G).

### 3.8 Inhibition of CXCR7 blocked the renoprotective effect of LESW

**Figure 7:**
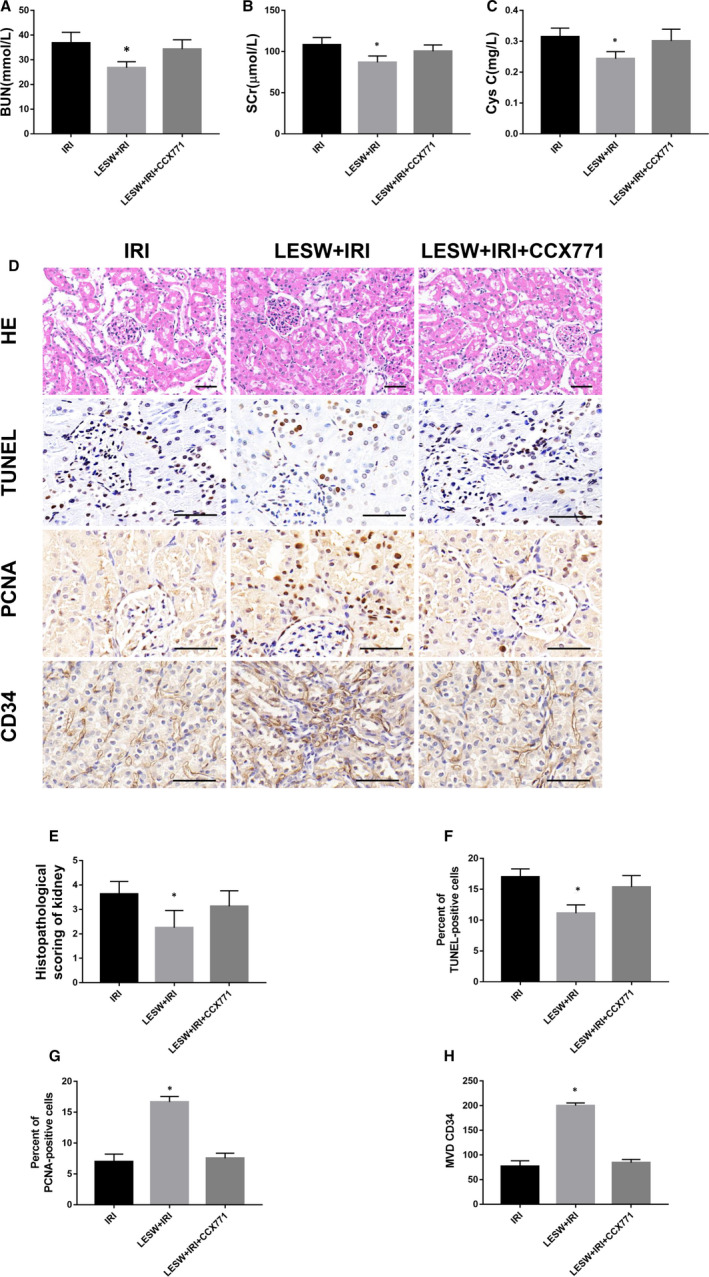
The renal protective effect of pretreatment with LESW was blocked by CCX771. A‐C, BUN, SCr, and Cys C were evaluated at 24 h after reperfusion in the three groups. D, Representative images of H&E, TUNEL, PCNA, and CD34 staining in the kidneys at 3 d after reperfusion in the three groups. E‐H, Histopathological scoring of kidney, Semi‐quantitative analysis of TUNEL‐positive cells, PCNA‐positive cells, and MVD. Scale bar = 50 μm. *P < .05 (IRI, LESW + IRI + CCX771 vs LESW + IRI). BUN, blood urea nitrogen; Cys C, cystatin C; EPCs, endothelial progenitor cells; H&E, haematoxylin and eosin; LESW, low‐energy shock wave; MVD, microvessel density; PCNA, proliferating cell nuclear antigen; SCr, serum creatinine; SDF‐1, stromal cell–derived factor‐1; TUNEL, terminal transferase‐mediated deoxyuridine triphosphate nick‐end‐labelling

As shown in Figure 7A‐C, LESW could obviously reduce the levels of kidney injury markers including BUN, SCr and Cys C in serum at 24 hours after reperfusion, whereas the protective role was suppressed after the administration of CCX771. Likewise, the renal tubular damage in LESW addressed kidneys also became severe after the inhibition of CXCR7 (Figure 7D,E). Besides, in the IRI kidneys treated with LESW, the proportion of TUNEL‐positive cells was significantly increased (Figure 7D,F), and the proportion of PCNA‐positive cells and MVD were remarkably decreased after CXCR7 blocking (Figure 7D,G,H).

### 3.9 Inhibition of CXCR7 prevented the recruitment of circulating EPCs

**Figure 8:**
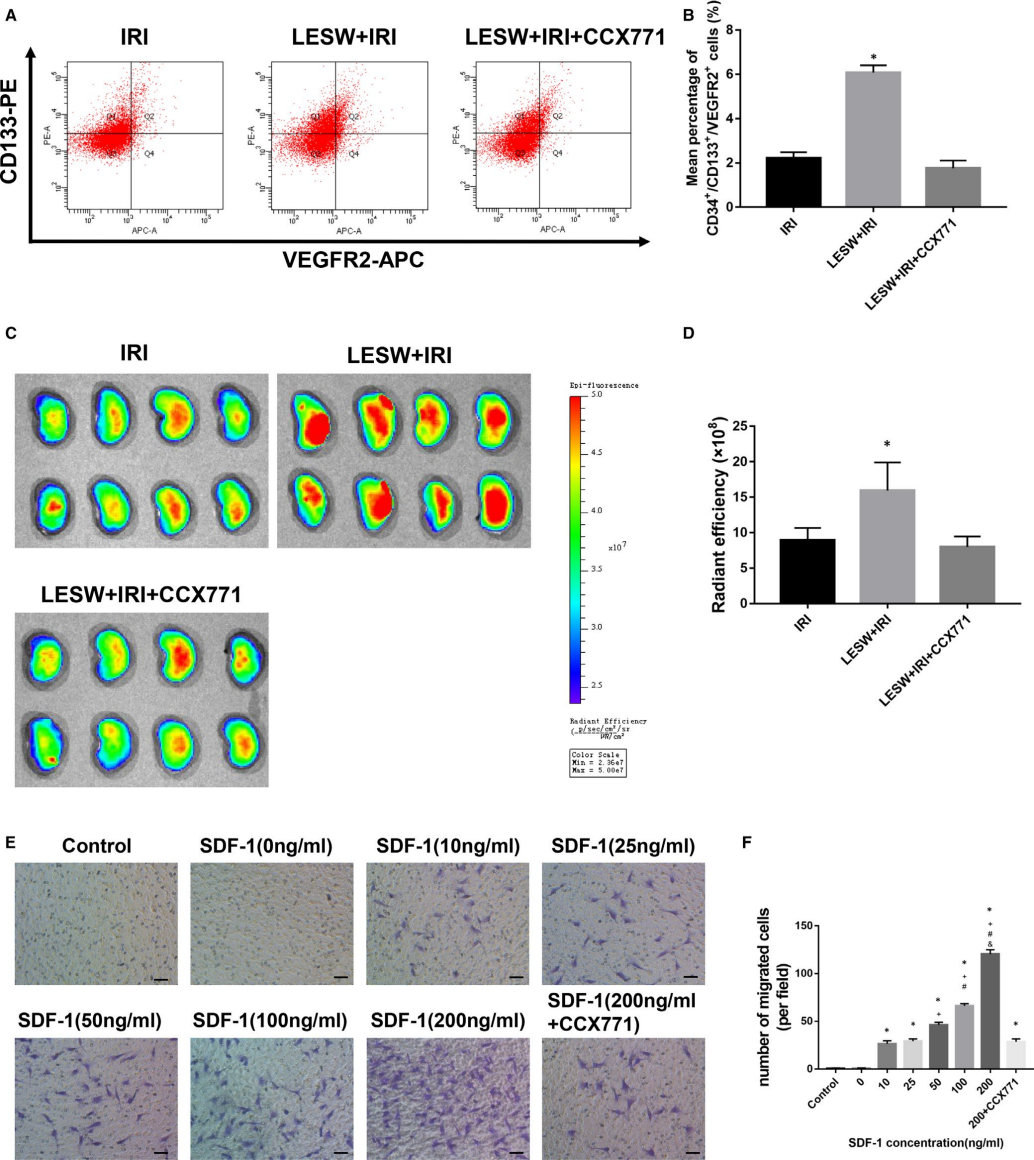
Inhibition of CXCR7 prevented the recruitment and migration of circulating endothelial progenitor cells (EPCs). A and B, Representative flow cytometry histograms of isolated EPCs in kidneys and quantitative analysis of CD34^+^/CD133^+^/VEGFR2^+^ cells in the three groups. *P < .05 (IRI, LESW + IRI + CCX771 vs LESW + IRI). C, Representative fluorescence images of kidneys detected by in vivo imaging system. D, Quantitative analysis of the retention of the Dil‐C18(5)‐DS labelled circulating EPCs in kidney at 24 h after reperfusion in the three groups. E, The migrated ability of EPCs following stimulating with SDF‐1 (0‐200 ng/mL) without or with CCX771. Representative images were shown. F, Quantitative analysis of circulating EPCs migration. Scale bar = 50 μm. *P < .05 (10, 25, 50, 100, 200, 200 + CCX771 vs control and 0); ^+^ *P* < .05 (50, 100, 200 vs 10, 25, and 200 + CCX771); ^#^ *P* < .05 (100, 200 vs 50); ^&^ *P* < .05 (200 vs 100). LESW, low‐energy shock wave; SDF‐1, stromal cell–derived factor‐1

We also inspected the total EPCs and labelled circulating EPCs in kidneys after the administration of CCX771. Results confirmed that the amount of total EPCs (CD133^+^/CD31^+^/VEGFR2^+^) was obviously decreased in rat kidneys treated with CCX771 (Figure 8A,B); meanwhile, the homing of circulating EPCs to impaired kidneys was also inhibited (Figure 8C,D).

### 3.10 Role of SDF‐1/CXCR7 pathway in circulating EPCs migration analysed by in vitro transwell migration assay

To further establish the promotion of SDF‐1/CXCR7 pathway in circulating EPCs migration, an in vitro transwell migration assay was conducted. With the increase of SDF‐1 concentration, the number of migrated circulating EPCs was accordingly increased; however, the facilitation was reversed by the CXCR7 antagonist CCX771 (Figure 8E,F).

## 4 DISCUSSION

Currently, LESW has received more and more attention for its safety and efficiency in a variety of diseases.^9^, ^26^, ^27^ A recent research confirmed that LESW could protect renal functions and ameliorate ischaemic acute kidney injury in rats.^28^ In consistent with these results, the present study demonstrated that LESW preconditioning could markedly reduce serum markers of kidney injury and attenuate tubular damage after renal IRI, meanwhile suppress cell apoptosis and promote cell proliferation and microvascular regeneration in kidney tissues. Moreover, by using fluorescence tracking, we firstly revealed that pretreatment with LESW could also enhance the homing of circulating EPCs to IRI kidneys, which might be achieved via SDF‐1/CXCR7 pathway probably.

High‐energy shock wave is initially introduced for extracorporeal lithotripsy in clinical settings.^29^ Recently, however, shock wave with low energy has been gradually promoted and practiced in functional urology.^30^ A previous study even confirmed that LESW could reduce the renal oxidative and inflammation caused by high‐energy shock wave lithotripsy.^31^ Furthermore, growing evidence also suggests that administration of LESW can alleviate substantial damage and improve functional recovery in various of organs or tissues, such as the heart,^8^ limb,^11^ bladder^32^ and penis.^9^ The beneficial effects might be attributed to activated cell proliferation,^33^ inhibition of inflammatory^34^ and neovascularization.^35^ Our findings also demonstrated that LESW could suppress cell apoptosis and increase cell proliferation and microvessel density to diminish renal tubular injury and accelerate renal recovery in rats subjected to IRI. What's more, LESW application did not induce significant damage in normal kidneys, which further validated the safety of LESW. Despite this, the underlying mechanism of the protective role of LESW has not been clearly identified.

Endothelial progenitor cells are a heterogeneous cell population circulating in the blood, which contribute to the neovascularization, endothelial repair, and vascular homeostasis.^36^ Prior study of our team revealed that ischaemic preconditioning could increase EPCs in IRI kidneys after partial nephrectomy in a rat model.^17^ Likewise, in this study, we also confirmed the increase content of EPCs in impaired kidneys after pretreated with LESW. Nevertheless, the source of increased EPCs, whether derived from the proliferation of renal progenitor cells or recruited from blood circulation, was still confused. Consequently, we isolated circulating EPCs, labelled them with the CM‐Dil and Dil‐C_18_(5)‐DS and tracked the marked cells using immunofluorescence and in vivo imaging. The results proved that pretreatment with LESW could facilitate the homing of circulating EPCs to the kidneys subjected to IRI, suggesting a part of increased EPCs in kidneys was recruited from circulation. We investigated the distribution of injected circulating EPCs in the kidney by an in vivo imaging system. Compared with traditional fluorescence section observation, the in vivo imaging system can integrally display the content and distribution of labelled EPCs in the kidney, and accurately analyse the amount of targeted cells.

SDF‐1 has been widely considered as a chemokine to mediate stem cells migration.^37^ CXCR7 is a 7‐transmembrane receptor, which was previously known as an orphan receptor until it was discovered as a receptor for SDF‐1. CXCR7 regulated the migration of EPCs to promote post‐stroke angiogenesis^38^ and also promoted homing of EPCs to ischaemic tissue.^21^ The present study demonstrated that LESW up‐regulated the expression of the SDF‐1 and CXCR7 in the IRI kidneys. Consistent with previous researches,^18^, ^39^ we further found that the expression of SDF‐1 in the kidneys was mainly increased at the epithelial cells of the distal tubules and collecting tubules after renal ischaemia reperfusion and LESW pretreatment. Hypoxia‐inducible factors (HIFs) are a family of transcription factors that mediates adaptive responses to hypoxia.^40^ It has been demonstrated that several HIFs can influence the expression of SDF‐1 through regulating the activation of transforming growth factor‐β (TGF‐β).^41^, ^42^ Notably, previous study also has confirmed that LESW can promote HIFs activation.^43^ Therefore, we speculate that the HIFs and TGF‐β pathway may be involved in the process of LESW regulated SDF‐1 expression in the IRI kidneys. CCX771, the inhibitor of CXCR7, was applied to further investigate the role of SDF‐1/CXCR7 pathway in the therapeutic effect of LESW. The results indicated that CCX771 efficiently suppressed the EPCs migration and blocked the renoprotective effect of LESW in these rats. These results supported that LESW enhanced circulating EPCs recruitment via activation of the SDF‐1/CXCR7 pathway in rats.

Several limitations of this study should be acknowledged when interpreting the results. Firstly, the optimal parameters of LESW for renal IRI treatment need further explore, including energy density, frequency, total number of impulses and pulse interval. Secondly, we only observed the changes of the kidneys in the short‐term and the long‐term effects of LESW were not inspected. Thirdly, the potential effects of LESW preconditioning on inherent renal progenitor cells were not clarified, which would be our future research direction.

## 5 CONCLUSION

The present study demonstrated that pretreatment with LESW could ameliorate renal injury and promote kidney repair after ischaemia reperfusion in a rat model. The protective effect of LESW might be attributed to the enhanced recruitment of circulating EPCs via regulating the SDF‐1/CXCR7 pathway. These results suggested that the non‐invasive LESW would be a promising strategy for renal IRI in future clinical practice.

## CONFLICT OF INTEREST

The authors declare no conflict of interest.

## AUTHOR CONTRIBUTION

**Jingyu Liu:** Conceptualization (equal); Data curation (equal); Investigation (equal); Methodology (equal); Writing‐original draft (equal); Writing‐review & editing (equal). **Quanliang Dou:** Conceptualization (equal); Data curation (equal); Investigation (equal); Methodology (equal); Writing‐original draft (equal); Writing‐review & editing (equal). **Changcheng Zhou:** Conceptualization (equal); Data curation (equal); Investigation (equal); Methodology (equal); Writing‐original draft (equal); Writing‐review & editing (equal). **Liuhua Zhou:** Resources (equal); Validation (equal); Visualization (equal). **Feng Zhao:** Resources (equal); Validation (equal); Visualization (equal). **Luwei Xu:** Resources (equal); Validation (equal); Visualization (equal). **Zheng Xu:** Formal analysis (equal); Software (equal); Supervision (equal). **Yuzheng Ge:** Formal analysis (equal); Software (equal); Supervision (equal). **Ran Wu:** Formal analysis (equal); Software (equal); Supervision (equal). **Ruipeng Jia:** Conceptualization (equal); Funding acquisition (equal); Methodology (equal); Project administration (equal); Writing‐original draft (equal); Writing‐review & editing (equal).

## Data Availability

The data that support the findings of this study are available from the corresponding author upon reasonable request.
